# Growth Factor-Dependent Proliferation of the Pancreatic β-cell Line βTC-tet: An Assay for β-cell Mitogenic Factors

**DOI:** 10.1080/15604280212526

**Published:** 2002

**Authors:** Dalit Milo-Landesman, Shimon Efrat

**Affiliations:** 1 Department of Human Genetics and Molecular Medicine Sackler School of Medicine Tel Aviv University Ramat Aviv, Tel Aviv 69978 Israel

## Abstract

The ability to expand normal pancreatic islet β cells in culture would significantly advance the prospects of cell therapy for diabetes. A number of growth factors can stimulate limited islet cell replication, however other factors may exist which are more effective β-cell-specific mitogens. The search for novel β-cell growth factors has been hampered by the lack of a β-cell-specific proliferation assay. We developed a simple and sensitive assay for β-cell growth factors based on a conditionally-transformed mouse β-cell line (βTC-tet). These cells express the SV40 T antigen (Tag) oncoprotein under control of the tetracycline (Tc) operon regulatory system. In the presence of Tc, Tag expression is tightly shut off and the cells undergo complete growth arrest. Here we show that the growth-arrested cells can proliferate in response to growth factors in the absence of Tag. Using this assay, a number of growth factors previously shown to be mitogenic to a mixed islet cell population were found to induce proliferation of pure β cells. We conclude that growth-arrested βTC-tet cells can be employed in a survey of factors from various sources for identifying novel factors with β-cell mitogenic activity.


*Int. Jnl. Experimental Diab. Res.*, 3:69-74, 2002
© 2002 Taylor and Francis
1560-4284/02 $12.00 + .00

### Growth Factor-dependent Proliferation of the Pancreatic ß-cell line ßTC-tet: An Assay for ß-cell Mitogenic Factors

DALIT MILO-LANDESMAN AND SHIMON EFRAT*

Department of Human Genetics and Molecular Medicine, Sackler School of Medicine, Tel Aviv University, Ramat Aviv, Tel Aviv 69978, Israel

*Received: August 30, 2001; In final form:October 28, 2001*

**The ability to expand normal pancreatic islet β cells in culture would significantly advance the prospects of cell therapy for diabetes. A number of growth factors can stimulate limited islet cell replication, however other factors may exist which are more effective β-cell-specific mitogens. The search for novel β-cell growth factors has been hampered by the lack of a β-cell-specific proliferation assay. We developed a simple and sensitive assay for β-cell growth factors based on a conditionally-transformed mouse β-cell line (βTC-tet). These cells express the SV40 T antigen (Tag) oncoprotein under control of the tetracycline (Tc) operon regulatory system. In the presence of Tc, Tag expression is tightly shut off and the cells undergo complete growth arrest. Here we show that the growth-arrested cells can proliferate in response to growth factors in the absence of Tag. Using this assay, a number of growth factors previously shown to be mitogenic to a mixed islet cell population were found to induce proliferation of pure β cells. We conclude that growth-arrested βTC-tet cells can be employed in a survey of factors from various sources for identifying novel factors with β-cell mitogenic activity.**

*Key words:* β-cell lines, cell proliferation, growth factors, mitogenicity, tetracycline-regulated gene expression

### INTRODUCTION

Beta-cell transplantation represents an attractive approach for replacement of the

*Corresponding author: tel: 972-3-640 7701; fax. 972-3-640 9950; e-mail: sefrat@post.tau.ac.il damaged β cells in type I diabetes. One major obstacle to β-cell therapy is the lack of an abundant cell supply. A number of rodent β-cell lines have been developed by oncogenic transformation, however this approach has proven difficult to adapt to human β cells. Development of efficient ways for in vitro expansion of normal islets would significantly advance the prospects of diabetes cell therapy. Adult islet cells are difficult to propagate in culture, although they maintain a proliferative capacity, as evidenced by a limited mitogenic response to a number of growth factors [1,2]. Members of the growth hormone family, in particular placental lactogen (PL) and prolactin (PRL), induce replication in neonatal rat islet cells [3]. These activities may be responsible for islet mass expansion in pregnancy. Significant mitogenic effects of hepatocyte growth factor (HGF) have been observed on human fetal and adult islets [4] and mouse islets [5]. Insulin–like growth factors (IGF) I and II, and platelet-derived growth factor, affect fetal rat islet cell growth [6,7]. IGF I has also been shown to stimulate replication of cultured rat insulinoma cells [8], and IGF II activates the growth of both normal [9] and transformed [10] mouse β cells *in vivo*. The Reg protein, produced by pancreatic acinar cells and regenerating islets, is another candidate for a β-cell growth factor [11]. These findings suggest that islet cells possess cell surface receptors and signal transduction pathways that render them responsive to these factors.

The search for novel β-cell growth factors has been hampered by the lack of a specific assay for β-cell proliferation. Normal islet cells are difficult to isolate reproducibly and in large numbers, and they contain various cell types, in addition to β cells. Conventional β-cell lines are not suitable for such an assay since their proliferation is largely independent of exogenous growth factors. In response to growth factors, most β-cell lines manifest a very limited increase in their proliferation [12]. We have developed a conditionally-transformed mouse β-cell line, denoted βTC-tet, by expression of SV40 T antigen (Tag) in β cells of transgenic mice under control of the tetracycline (Tc) operon regulatory system [13]. In the presence of Tc, Tag expression is tightly shut off and the cells undergo complete growth arrest. Here we show that the growth-arrested cells manifest a potent proliferative response to a number of growth factors, and describe a simple and sensitive assay that can serve to screen for novel factors with potential β-cell mitogenic activity.

### MATERIALS AND METHODS

### *Cell Culture*

The generation of the βTC-tet line has been previously described [13]. Cells were grown in Dulbecco's modified Eagle's medium (DMEM) containing 25 mM glucose and supplemented with 15% horse serum, 2.5% fetal bovine serum (FBS), 2 mM L-glutamine, 100 U per ml penicillin, and 100 μg per ml streptomycin (all media supplies were from Beit-Haemek Biological Industries, Israel). Growth arrest was induced by including 1 μg per ml Tc (Sigma Chemicals) in the culture medium. All the experiments described here were performed with cells at passages 29-40.

### *Cell proliferation assay*

βTC-tet cells were plated into wells of 96-well plates at 2-4X$10^4$ cells per well in the presence of Tc. Ten to 14 d later the growth-arrested cells were washed twice with PBS and preincubated in DMEM containing L-glutamine and antibiotics in the absence of serum and in the presence of Tc. Twenty four hours later the medium was removed, and replaced with the same medium containing the indicated growth factors (Sigma Chemicals), for the indicated period of time. During the last 16 h of incubation each well was pulsed with 1 μCi [methyl-

[3]H]thymidine (ICN; 67 Ci per mmol). The cells were then lysed in water using a Dynatec cell harvester (Automash 2000), and the DNA was retained on a glass fiber filter (Whatman). The radioactivity incorporated into DNA was quantitated with a scintillation counter. For cell counting, $2X10^5$ cells were plated in wells of 24-well plates. Following a 72-h treatment with growth factors as above, the cells were stained with trypan blue and counted in a hemocytometer. Each condition was assayed in at least 3 replicates.

### RESULTS AND DISCUSSION

βTC-tet cells were shown to depend on Tag expression for proliferation [13]. To determine whether growth-arrested βTC-tet cells can be induced to resume cell division in the absence of Tag, growth-arrested cells were incubated in the presence of a number of growth factors with documented mitogenic effects on both rodent and human fetal, neonatal, or adult islet cells. The factors were assayed at physiological concentrations previously shown to be mitogenic to islet cells, as well as at other concentrations. The serum was excluded from the assay medium to eliminate potential background from growth factors present in serum. As shown in Figure 1, four growth factors (EGF, PRL, PL, and IGF II) manifested a robust mitogenic effect on growth-arrested βTC-tet cells following a 3-d incubation with the factors in DMEM without serum. The effect was in the range of 6-8-fold increase in thymidine incorporation over that of growth-arrested cells incubated in the absence of growth factors. Four other growth factors tested (HGF, growth hormone, IGF I, and acidic fibroblast growth factor) manifested a considerable but more modest mitogenic effect, in the 2-4-fold range.

The duration of the thymidine pulse (16 h) was established empirically, to maximize the signal. The optimal timing of the pulse was found to be approximately 3 days from the onset of treatment with the growth factors (data not shown). The doubling time of βTC-tet cells in the absence of Tc is close to 4 days. The entrance into cell cycle from a growth-arrested state may involve an additional lag period. There was a distinct increase in thymidine incorporation when the cells were harvest-

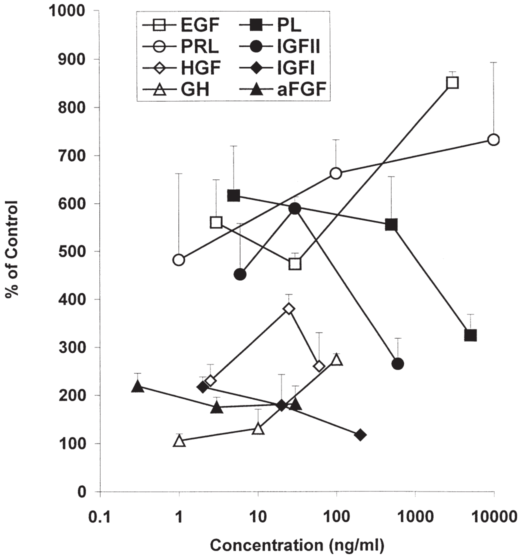

FIGURE 1
Dose-dependent proliferation of βTC-tet cells in response to growth factors. βTC-tet cells were incubated in complete growth medium for 10-14 d in the presence of Tc to induce growth arrest. They were then incubated for 72 h in the absence of serum and presence of Tc and the indicated growth factors. During the last 16 h of incubation the cells were then pulsed with [3H]thymidine, and the radioactivity incorporated into DNA was quantitated. The fold stimulation was calculated by dividing the cpm obtained with each growth factor by the cpm incorporated into cells incubated in the presence of Tc and the absence of growth factors. Values represent mean ± SEM of 3 separate experiments. Typical cpm values obtained in this assay were about $5X10^2$ cpm for cells growth arrested in the presence of Tc and not treated with growth factors, and $1.5X10^4$ cpm for cells incubated in regular growth medium without Tc (both in the presence or absence of serum). IGF I and II, human recombinant insulin-like growth factor I and II; EGF, mouse epidermal growth factor; PRL, sheep prolactin; PL, human placental lactogen; GH, human growth hormone; aFGF, bovine acidic fibroblast growth factor; HGF, human recombinant hepatocyte growth factor.

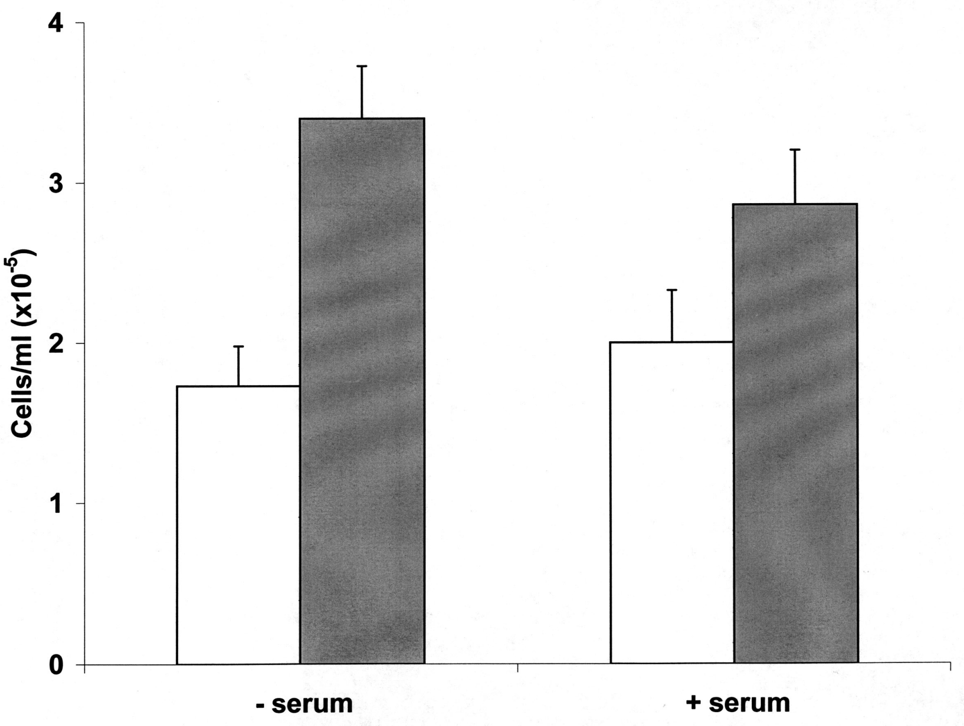

FIGURE 2
Effect of EGF on cell number in growth-arrested βTC-tet cells. βTC-tet cells were incubated in complete growth medium for 10 d in the presence of Tc to induce growth arrest. They were then incubated for 72 h in the presence of Tc, and in the absence (open bars) or presence (closed bars) of 1 μg per ml EGF, in either complete or serum-free medium, as indicated. Cells in triplicate wells were counted by hemocytometer. Values are mean ± SEM.

ed following a 72-h exposure to the growth factors, as compared to 48 h or 96 h. This may reflect the possibility that the growth-arrested cells respond synchronously when stimulated by a mitogenic activity, and there are more cells in S-phase between 56-72 h following stimulation, compared with 32-48 h or 80-96 h following stimulation.

To confirm that the increase in DNA synthesis reflects an increase in cell number, growth-arrested cells were treated with the growth factor that gave the highest stimulation of DNA synthesis, EGF at 1 μg per ml, and the increase in cell number was quantitated. As seen in Figure 2, the cell number doubled following a 3-d treatment, indicating that the vast majority of cells was stimulated to divide.

The serum included in the complete growth medium likely contains some of the growth factors shown to be mitogenic to growth-arrested βTC-tet cells. Nevertheless, these cells can undergo growth arrest in medium with serum. It is possible that the concentration of these factors in the serum is too low to stimulate the proliferation of growth-arrested cells, or that they may be complexed with binding proteins that influence their availability for biological activity, as has been demonstrated for the IGFs [14]. In contrast, in the serum-free assay medium, addition of purified growth factors in the absence of the binding proteins reveals a more pronounced mitogenic effect (Figure 2).

We have routinely used DMEM containing 25 mM glucose for propagation of βTC lines, including βTC-tet cells, since culture in low-glucose medium often leads to cell dedifferentiation in these lines (S. Efrat , M. Surana, and N. Fleischer, unpublished results). This long-term exposure to high glucose concentrations does not seem to result in glucose toxicity, as judged by the normal insulin production and glucose sensing maintained in βTC-tet cells for over 60 passages [15]. However, these culture conditions might have been expected to confuse the proliferation assay, since the glucose concentration used could itself have a stimulatory effect on cell replication. In practice, glucose does not appear to have a significant mitogenic effect in βTC-tet cells, as demonstrated by the fact that growth arrest of these cells occurs in the presence of 25 mM glucose. The low baseline of the assay, which is also performed in DMEM with 25 mM glucose, is a further confirmation of the negligible mitogenic effect of glucose in this system.

The results obtained with the βTC-tet cells are mostly consistent with the reported effects of the tested growth factors on normal islet cells. Thus, the mitogenic effects of these factors on a mixed population of islet cells are largely confirmed here with pure β cells. The limited activity of growth hormone, compared with the larger effect of PRL, is in agreement with results obtained with homologous hormones [3]. Although the heterologous human growth hormone can stimulate rat islet cell replication [16], most likely through the PRL receptor, it has only a modest effect on mouse islet cells [17]. The relatively lower mitogenic effect of IGF I in this assay, as compared with that of IGF II, is inconsistent with previous results that showed similar effects of both factors on fetal rat islets [7], and with the action of both factors through the IGF I receptor. It is possible that βTC-tet cells express IGF binding proteins that preferentially neutralize IGF I activity. The considerable mitogenic effect of HGF (4-fold) confirms previous results obtained with human islets [4]. Lefebvre et al. [18] suggested that the HGF effect may be due primarily to its mitogenicity to pancreatic duct cells, which contaminate islet preparations, rather than to its effect on β-cell proliferation. The findings presented here support a direct effect of HGF on murine β cells. In this context the results with HGF demonstrate the advantage of an assay based on a population of pure β cells, over the use of the mixed cell population in primary islets, for assessment of β-cell growth factor activities.

These results demonstrate that growth-arrested βTC-tet cells maintain a proliferative capacity in the absence of Tag and can re-enter the cell cycle given appropriate stimuli in a manner similar to that of normal islet β cells. Thus they constitute an authentic model for assessing the effect of growth factors on normal β-cell proliferation. These cells can replace isolated islets as an abundant and pure β-cell population for screening agents from various sources for potential β-cell mitogenic activities. The thymidine incorporation assay in microtiter plates is a simple, sensitive, and reproducible assay which is well-suited for a large-scale survey and for monitoring factor purification from a crude source. Mitogenic activities identified by this assay can then be tested on non-transformed β cells from isolated animal or human islets, to confirm their usefulness for expansion of normal islet β cells.

***Acknowledgements***

This work was funded by the Israel Science Foundation, The Endowment Fund for Basic Research in Life Sciences, Charles H. Revson Foundation. This work was performed in partial fulfillment of the requirements for a Ph.D. degree of Dalit Milo-Landesman, Sackler School of Medicine, Tel Aviv University.

### REFERENCES

1. Nielsen, J.H., Galsgaard, E.D., Moldrup, A., Friedrichsen, B.N., Billestrup, N., Hansen, J.A., Lee, Y.C. and Carlsson, C. (2001). Regulation of beta-cell mass by hormones and growth factors, *Diabetes*, **50**, Suppl. 1, S25-S29.
2. Garcia-Ocana, A., Vasavada, R.C., Takane, K.K., Cebrian, A., Lopez-Talavera, J.C. and Stewart, A.F. (2001). Using beta-cell growth factors to enhance human pancreatic islet transplantation, *J. Clin. Endocrinol. Metab.*, **86**, 984-988.
3. Brelje, T.C., Scharp, D.W., Lacy, P.E., Orgen, L., Talamantes, F., Robertson, M., Friesen, H.G. and Sorenson, R.L. (1993). Effect of homologous placental lactogens, prolactins, and growth hormones on islet β-cell division and insulin secretion in rat, mouse and human islets: implication for placental lactogen regulation of islet function during pregnancy, *Endocrinology*, **132**, 879-887.
4. Beattie, G.M., Rubin, J.S., Mally, M.I., Otonkoski, T. and Hayek, A. (1996). Regulation of proliferation and differentiation of human fetal pancreatic islet cells by extracellular matrix, hepatocyte growth factor, and cell-cell contact, *Diabetes,* **45**,1223-1228.
5. Garcia-Ocana, A., Takane, K.K., Syed, M.A., Philbrick, W.M., Vasavada, R.C. and Stewart A.F. (2000). Hepatocyte growth factor overexpression in the islet of transgenic mice increases beta cell proliferation, enhances islet mass, and induces mild hypoglycemia. *J. Biol. Chem.,* **275**, 1226-1232.
6. Swenne, I., Heldin, C-H., Hill, D.J. and Hellerstrom, C. (1988). Effects of platelet-derived growth factor and somatomedin-c/insulin-like growth factor I on the deoxyribonucleic acid replication of fetal rat islets of Langerhans in tissue culture, *Endocrinology*, **122**, 214-218.
7. Hogg, J., Han, V.K.M., Clemmons, D.R. and Hill, D.J. (1993). Interactions of nutrients, insulin-like growth factors (IGFs), and IGF-binding proteins in the regulation of DNA synthesis in isolated fetal rat islets of Langerhans, *J. Endocrinol.*, **138**, 410-412.
8. Hugl, S.R., White, M.F. and Rhodes, C.J. (1998). Insulin-like growth factor I (IGF-I)-stimulated pancreatic beta-cell growth is glucose-dependent. Synergistic activation of insulin receptor substrate-mediated signal transduction pathways by glucose and IGF-I in INS-1 cells, *J. Biol. Chem.* **273**, 17771-17779.
9. Petrik, J., Pell, J.M., Arany, E., McDonald, T.J., Dean, W.L., Reik, W. and Hill, D.J. (1999). Overexpression of insulin-like growth factor-II in transgenic mice is associated with pancreatic islet cell hyperplasia, *Endocrinology,* **140**, 2353-2363.
10. Christofori, G., Naik, P. and Hanahan, D. (1994). A second signal supplied by insulin-like growth factor II in oncogene-induced tumorigenesis, *Nature,* **369**, 414-418.
11. Watanabe, T., Yonemura, Y., Yonekura, H., Suzuki, Y., Miyashita, H., Sugiyama, K., Moriizumi, S., Unno, M., Tanaka, O., Kondo, H., Bone, A.J., Takasawa, S. and Okamoto, H. (1994). Pancreatic beta-cell replication and amelioration of surgical diabetes by reg protein, *Proc. Natl. Acad. Sci. USA,* **91**, 3589-3592.
12. Houtari, M-A., Palgi, J. and Otonkoski, T. (1998). Growth factor-mediated proliferation and differentiation of insulin-producing INS-1 and RINm5F cells: identification of betacellulin as a novel β-cell mitogen, *Endocrinology*, **139**, 1494-1499.
13. Efrat, S., Fusco-DeMane, D., Lemberg, H., Emran, O.A. and Wang, S. (1995). Conditional transformation of a pancreatic β-cell line derived from transgenic mice expressing a tetracycline-regulated oncogene, *Proc. Natl .Acad. Sci.USA,* **92**, 3576-3580.
14. Jones, J.I. and Clemmons, D.R. (1995). Insulin-like growth factors and their binding proteins: biological actions, *Endocrine Rev.,* **16**, 3-34.
15. Fleischer, N., Chen, C., Surana, M., Leiser, M., Rossetti, L., Pralong, W., Efrat, S. (1998). Functional analysis of a conditionally-transformed pancreatic β-cell line, *Diabetes,* **47**, 1419-1425.
16. Nielsen, J.H., Linde, S., Welinder, B.S., Billestrup, N. and Madsen, O.D. (1989) Growth hormone is a growth factor for the differentiated pancreatic b cell. *Mol. Endocrinol.,* **3**, 165-173.
17. Nielsen, J.H. (1982). Effects of growth hormone, prolactin, and placental lactogen on insulin content and release, and deoxyribonucleic acid synthesis in cultured pancreatic islets, *Endocrinology,* **110**, 600-606.
18. Lefebvre, V.H., Otonkoski, T., Ustinov, J., Huotari, M-A., Pipeleers, D.G. and Bouwens, L. (1998). Culture of adult human islet preparations with hepatocyte growth factor and 804G matrix is mitogenic for duct cells but not for β-cells, *Diabetes,* **47**, 134-137.

